# Differential Contribution of Gap Junctions to the Membrane Properties of ON- and OFF-Bipolar Cells of the Rat Retina

**DOI:** 10.1007/s10571-020-00845-y

**Published:** 2020-04-22

**Authors:** Rémi Fournel, Espen Hartveit, Margaret Lin Veruki

**Affiliations:** Department of Biomedicine, University of Bergen, Jonas Lies vei 91, 5009 Bergen, Norway

**Keywords:** Cone bipolar cells, Connexin 36, Connexin 45, Electrical coupling, Gap junctions, Retina

## Abstract

Gap junctions are ubiquitous within the retina, but in general, it remains to be determined whether gap junction coupling between specific cell types is sufficiently strong to mediate functionally relevant coupling via electrical synapses. From ultrastructural, tracer coupling and immunolabeling studies, there is clear evidence for gap junctions between cone bipolar cells, but it is not known if these gap junctions function as electrical synapses. Here, using whole-cell voltage-clamp recording in rat (male and female) retinal slices, we investigated whether the gap junctions of bipolar cells make a measurable contribution to the membrane properties of these cells. We measured the input resistance (*R*_N_) of bipolar cells before and after applying meclofenamic acid (MFA) to block gap junctions. In the presence of MFA, *R*_N_ of ON-cone bipolar cells displayed a clear increase, paralleled by block of the electrical coupling between these cells and AII amacrine cells in recordings of coupled cell pairs. For OFF-cone and rod bipolar cells, *R*_N_ did not increase in the presence of MFA. The results for rod bipolar cells are consistent with the lack of gap junctions in these cells. However, for OFF-cone bipolar cells, our results suggest that the morphologically identified gap junctions between these cells do not support a junctional conductance that is sufficient to mediate effective electrical coupling. Instead, these junctions might play a role in chemical and/or metabolic coupling between subcellular compartments.

## Introduction

Gap junctions are seemingly ubiquitous components of neural circuits throughout the vertebrate retina (reviewed by Völgyi et al. 2013). As such, the retina has long been a model system for studying how gap junctions mediate electrical coupling and how they play an important role in signaling, plasticity, and neurodegeneration (reviewed by Trenholm and Awatramani 2017; O'Brien and Bloomfield 2018). For a few of these gap junctions, a clear physiological role in the processing of visual signals has been identified. For example, homologous coupling between cone photoreceptors (DeVries et al. 2002) and rod photoreceptors (Li et al. 2012) enhance the signal-to-noise ratio, and heterologous gap junctions between AII amacrine and ON-cone bipolar cells are thought to mediate signal transfer between ON- and OFF-pathways under both scotopic (reviewed by Bloomfield and Dacheux 2001) and photopic conditions (Manookin et al. 2008; Münch et al. 2009; Kuo et al. 2016). However, a clear functional role for most gap junctions in the retina remains to be determined.

A particularly interesting case of retinal gap junctions for which there is strong morphological evidence, but virtually no information regarding a putative functional role, are the gap junctions between different types of cone bipolar cells. There are roughly 10–12 types of cone bipolar cells in mammalian retina and as many as 20 different types in non-mammalian retina. These types are generally divided into ON-types that depolarize at light onset and OFF-types that depolarize at light offset (reviewed by Euler et al. 2014). Ultrastructural studies in teleost and mammalian retina have found evidence for gap junctions between dendrites of both OFF- and ON-cone bipolar cells (Raviola and Gilula 1975; Umino et al. 1994), between axon terminals of OFF-cone bipolar cells (Kolb 1979; Marc et al. 1988; Jacoby and Marshak 2000; Tsukamoto and Omi 2015), and between axon terminals of ON-cone bipolar cells (Cohen and Sterling 1990; Tsukamoto and Omi 2017). These electron-microscopic studies are supported by light-microscopic studies of immunolabelling for connexin 36 (Cx36) and connexin 45 (Cx45). Cx36 has been found at the dendrites of OFF-cone bipolar cells (mouse: Feigenspan et al. 2004; macaque: O'Brien et al. 2012; human: Kántor et al. 2016; Kántor et al. 2017) and at the axon terminals of ON-cone bipolar cells (mouse: Han and Massey 2005; Lin et al. 2005; Dedek et al. 2006). In mouse, Cx45 has been found at both the dendrites and axon terminals of OFF-cone bipolar cells (Lin et al. 2005; Hilgen et al. 2011) and the axon terminals of ON-cone bipolar cells (Dedek et al. 2006). These ultrastructural and immunolabeling studies are complemented by evidence for tracer coupling between OFF-cone bipolar cells in rabbit retina (Mills 1999) and between both ON- and OFF-cone bipolar cells in tiger salamander retina (Zhang and Wu 2009). In addition, both tracer coupling and electrical coupling (using dual patch-clamp recording) has been observed between Mb1 bipolar cells in goldfish retina (Arai et al. 2010). Finally, electrical coupling between adjacent bipolar cells was observed with paired intracellular recordings in carp retina (Kujiraoka and Saito 1986). Taken together, there is substantial evidence that both ON- and OFF-cone bipolar cells make gap junction contacts with other cells of the same class, and that this is a common circuit motif in the vertebrate retina. In contrast, there are no reports of gap junctions between rod bipolar cells (e.g., Strettoi et al. 1990).

The different types of bipolar cells are thought to form parallel channels encoding distinct stimulus properties such as contrast, chromatic features, and temporal properties (reviewed by Euler et al. 2014), and it is not at all clear how gap junctions between bipolar cells are consistent with this view. Thus, the question arises as to whether all morphologically identified gap junctions correspond physiologically to electrical synapses. Arguably, the most direct approach to answer this question would be to perform dual recordings from pairs of visually identified cone bipolar cells, ideally in genetically modified animals with fluorescently labeled cells that could be targeted for recording. Even when such animals become available, the electrical coupling between ON-cone bipolar cells and AII amacrine cells (Veruki and Hartveit 2002b) could confound measurements for ON-cone bipolar cells, limiting the approach to OFF-cone bipolar cells. An alternative approach, that we present here, is to perform electrophysiological recording from single neurons and examine if pharmacological block of gap junction-mediated electrical coupling increases the input resistance (*R*_N_) of the cells (cf. Alcamí and Pereda 2019). For both AII amacrine cells (Veruki et al. 2010) and A17 amacrine cells (Elgueta et al. 2018), the gap junction blocker meclofenamic acid (MFA) evokes an increase of *R*_N_, fully consistent with the simultaneously observed block of junctional conductance (in paired recordings from electrically coupled cells) and the evidence for homologous coupling between AII (Kolb and Famiglietti 1974; Vaney 1991; Strettoi et al. 1992; Veruki and Hartveit 2002a) and A17 amacrines (Li et al. 2002; Grimes et al. 2014; Elgueta et al. 2018). Similar observations have also been made for Golgi cell interneurons in the cerebellar cortex (Szoboszlay et al. 2016). Thus, if MFA evokes a similar increase of *R*_N_ for other neurons with morphological evidence for gap junctions, it is reasonable to assume that the increase of *R*_N_ corresponds to a reduction of the junctional conductance between electrically coupled cells. Here we investigated the effect of MFA on the *R*_N_ of bipolar cells in the rat retina. For ON-cone bipolar cells, *R*_N_ displayed a clear increase, as expected for cells with gap junction-mediated electrical coupling. For rod bipolar cells, *R*_N_ did not increase in the presence of MFA, consistent with their lack of gap junctions. Surprisingly, given the substantial morphological evidence for the presence of gap junctions between OFF-cone bipolar cells, *R*_N_ for these cells did not increase following application of MFA. Our results suggest that the gap junctions between OFF-cone bipolar cells do not support consequential electrical coupling.

## Materials and Methods

### Retinal Slice Preparation

General aspects of the methods have previously been described in detail (Hartveit 1996). The use of animals in this study was carried out under the approval of and in accordance with the regulations of the Animal Laboratory Facility at the Faculty of Medicine at the University of Bergen (accredited by AAALAC International). Male and female albino rats (Wistar HanTac, bred in-house or purchased from Taconic Bioscience, Denmark; 4–7 weeks postnatal) had ad libitum access to food and water and were kept on a 12/12 light/dark cycle. Animals were deeply anaesthetized with isoflurane (IsoFlo vet 100%; Abbott Laboratories) in 100% O_2_ and killed by cervical dislocation. After removing the eyes and dissecting out the retinas, retinal slices were cut by hand with a curved scalpel blade at a thickness of ~ 100 to ~ 150 µm. In some experiments, the slices were visualized with a conventional, upright microscope (BX51WI; Olympus) with a × 60 (0.9 NA) or × 40 (0.8 NA) water immersion objective (Olympus). Infrared (IR) video microscopy was performed with an IR-sensitive analog CCD camera (VX55; TILL Photonics) and either differential interference contrast (IR-DIC) or Dodt gradient contrast (IR-DGC; Luigs & Neumann) for contrast enhancement. In other experiments, the slices were visualized using a custom-modified "Movable Objective Microscope" (MOM; Sutter Instrument) with a × 20 water immersion objective (XLUMPLFL; 0.95 NA; Olympus) and IR-DGC videomicroscopy. The cell bodies of the recorded cells were generally located 15–30 µm below the surface of the slice. Electrophysiological recording and imaging were carried out at room temperature (22–25 °C). Anesthesia, dissection, and preparation of slices were done under normal room illumination. During recording at conventional upright microscopes, the room lights were dimmed moderately for the purpose of observing monitor displays better. During recording at the MOM for multiphoton excitation (MPE) microscopy, the room lights were dimmed and the preparation was located in a cage covered by black cloth. Taken together, we consider the slices to be light adapted (cf. Veruki and Hartveit 2002b).

### Solutions and Drugs

The extracellular perfusing solution was continuously bubbled with 95% O_2_–5% CO_2_ and had the following composition (in mM): 125 NaCl, 25 NaHCO_3_, 2.5 KCl, 2.5 CaCl_2_, 1 MgCl_2_, 10 glucose, pH 7.4 (osmolarity ~ 300 mOsm). For single recordings of bipolar cells, the recording pipettes were filled with (in mM): 125 K-gluconate, 5 KCl, 8 NaCl, 10 Hepes, 0.2 EGTA, 4 MgATP, 0.4 Na_3_GTP (pH adjusted to 7.3 with KOH). For visualization of the cells by fluorescence microscopy after the recording, the pipette solution contained Alexa Fluor 594 (40 or 60 µM). All Alexa Fluor dyes were used as hydrazide sodium salts (Invitrogen/Thermo Fisher Scientific). In experiments with simultaneous dual recording from electrically coupled cells using low-resistance recording pipettes and conventional patch-clamp amplifiers (see below), the pipettes for AII amacrine cells were filled with (in mM): 125 K-gluconate, 8 NaCl, 10 Hepes, 1 CaCl_2_, 5 EGTA, 4 MgATP, 2*N*-(2,6-dimethylphenylcarbamoylmethyl)triethylammonium bromide (QX-314), 0.1 Alexa Fluor 488 (pH adjusted to 7.3 with KOH), and the pipettes for bipolar cells were filled with (in mM): 130 KCl, 8 NaCl, 10 Hepes, 1 CaCl_2_, 5 EGTA, 4 MgATP, 0.04 Alexa Fluor 594 (pH adjusted to 7.3 with KOH). In similar experiments using higher-resistance pipettes and switch-clamp amplifiers, the pipettes for both AII amacrine and bipolar cells were filled with (in mM): 125 K-gluconate, 5 KCl, 8 NaCl, 10 Hepes, 0.2 EGTA, 4 MgATP (pH adjusted to 7.3 with KOH). For visualization by fluorescence microscopy, this pipette solution contained Lucifer yellow (1 mg/ml).

The theoretical liquid junction potential (the potential of the extracellular solution relative to that of the intracellular solution) was calculated with the software program JPCalcW (Molecular Devices, Sunnyvale, CA, USA) and in recordings with conventional patch-clamp amplifiers (see below), all membrane holding potentials (*V*_hold_) were automatically corrected for the liquid junction potential on-line by the data acquisition software (Patchmaster; HEKA Elektronik, Lambrecht/Pfalz, Germany). For other recordings, we corrected the membrane holding potentials for the liquid junction potential during off-line analysis.

Drugs were added directly to the extracellular solution used to perfuse the slices. The concentrations of drugs were as follows (µM; supplier Tocris Bioscience, Bristol, UK; unless otherwise noted): 10 bicuculline methochloride, 1 strychnine (Research Biochemicals Inc., Natick, MA, USA), 10 6-cyano-7-nitroquinoxaline-2,3-dione (CNQX), 50 (1,2,5,6-tetrahydropyridin-4-yl)methylphosphinic acid (TPMPA), 0.3 or 1 tetrodotoxin (TTX), 50 4-ethylphenylamino-1,2-dimethyl-6-methylaminopyrimidinium chloride (ZD7288), and 20 (RS)-3-(2-carboxypiperazin-4-yl)-propyl-1-phosphonic acid (CPP). To block electrical coupling via gap junctions, we added 100 µM 2-[(2,6-dichloro-3-methylphenyl)amino]benzoic acid sodium salt (MFA; Sigma-Aldrich) to the extracellular solution (Veruki and Hartveit 2009). Solutions were either made up freshly for each experiment or were prepared from concentrated aliquots stored at − 20 °C.

### Electrophysiological Recording and Data Acquisition

Patch pipettes were pulled from thick-walled borosilicate glass (outer diameter, 1.5 mm; inner diameter, 0.86 mm; Sutter Instrument, Novato, CA, USA). In all single-cell recordings, the pipettes were coated with Parafilm (American National Can; Greenwich, CT, USA) to reduce their effective capacitance.

For electrophysiological recording, we used the whole-cell configuration of the patch-clamp technique, either with conventional patch-clamp amplifiers (continuous single-electrode voltage-clamp; CSEVC; EPC10-triple or EPC10-USB-dual; HEKA Elektronik) or with discontinuous (switched) single-electrode voltage-clamp (DSEVC) amplifiers (SEC-05LX-BF; npi Electronic, Tamm, Germany). All single-cell recordings were performed with CSEVC amplifiers. Dual, simultaneous recordings between electrically coupled cells were either performed with a CSEVC amplifier or with two DSEVC amplifiers. All amplifiers were controlled by Patchmaster software (HEKA Elektronik).

For recordings with CSEVC amplifiers, we used lower-resistance pipettes where the open-tip resistance ranged from ~ 7 to ~ 10 MΩ when filled with intracellular solution. After establishing a GΩ-seal, currents caused by the recording electrode capacitance were automatically measured and neutralized by the amplifier (*C*_fast_ function of Patchmaster software). After breaking into the cell, currents caused by the cell membrane capacitance were partially neutralized by the amplifier (*C*_slow_ function of Patchmaster software). Signals were low-pass filtered (analog 3- and 4-pole Bessel filters in series) with a corner frequency (− 3 dB) at 1/5 of the inverse of the sampling interval (typically 50 µs). Simultaneous, dual recordings of electrically coupled cell pairs with CSEVC amplifiers were performed as described for single cells, but when we recorded currents to estimate a cell’s membrane capacitance, the voltage-clamp stimuli were sent simultaneously to both amplifiers to eliminate junctional currents between the two cells. Dual recordings with DSEVC amplifiers were performed as described in earlier studies from our laboratory (Veruki and Hartveit 2009; Veruki et al. 2010).

Whole-cell voltage-clamp recording with CSEVC amplifiers was used to sample current responses used during offline analysis to estimate *R*_s_ and *R*_N_. For sampling capacitative current transients, the *C*_slow_ capacitance neutralization circuitry was disabled and the time constant of the internal stimulus filter was set to 2 µs. The sampling interval was set to 10 µs and before sampling, signals were low-pass filtered (analog 3-pole Bessel filter) with a corner frequency (− 3 dB) of 30 kHz. Current responses were evoked by 20 ms long voltage pulses of alternating amplitudes of ± 5 or − 10 mV from the holding potential of − 60 mV. Groups of 100 responses were acquired at intervals of 100 ms and averaged online. When we sampled other current responses, the *C*_slow_ capacitance neutralization circuitry was re-enabled and the time constant of the internal stimulus filter was set to 20 µs.

### Image Acquisition for MPE Microscopy and Wide-Field Fluorescence Microscopy

For MPE microscopy, fluorescence from neurons filled with Alexa 594 was imaged with the MOM equipped with a mode-locked Ti:sapphire laser (Mai Tai DeepSee; Spectra-Physics) tuned to 810 nm (for additional details, see Zandt et al. 2017). An image stack was acquired as a series of optical sections (1024 × 1024 pixels) with *XY* pixel size ~ 70 to ~ 80 nm (depending on the magnitude of the digital zoom factor) and collected at a focal plane interval of 0.4 µm. For each image stack, we acquired two channels and at each focal plane two images were averaged on-line. The first channel sampled the fluorescence light as described above. The second channel was used for IR laser scanning gradient contrast imaging (IR-LSGC; Yasuda et al. 2004) and sampled the forward-scattered IR laser light after it passed the substage condensor and a Dodt gradient contrast tube (Luigs & Neumann). MPE microscopy and image acquisition were controlled by ScanImage software (version 3.8.1; Pologruto et al. 2003) running under Matlab (The Mathworks).

In the experiments with dual recording of electrically coupled cells using CSEVC amplifiers, we used wide-field fluorescence microscopy to acquire image stacks of the cells filled with Alexa 594 via the patch pipette (TILLvisION system with a Polychrome V light source and an Imago QE cooled CCD camera; TILL Photonics; for a detailed description, see Castilho et al. 2015). Deconvolution of Z stacks acquired by MPE or wide-field fluorescence microscopy for morphology and generation of maximum intensity projections was performed as described in Zandt et al. (2017).

### Data Analysis and Presentation

To estimate the steady-state *G*_j_ between two electrically coupled cells, we used current responses obtained with dual voltage-clamp recording. For the calculations, we assumed an equivalent-circuit model (see, e.g., Veruki et al. 2010). For the dual recordings with DSEVC amplifiers, we assumed that *R*_s_ was effectively zero. For this case, the junction current (*I*_j_) corresponds to the current evoked in the postsynaptic cell when the presynaptic cell is stepped from *V*_hold_ and *G*_j_ can be calculated directly from Ohm's law (Veruki et al. 2010; for a detailed analysis, see Hartveit and Veruki 2010) according to Eq. (1) for voltage pulses applied to cell *a* of a pair and according to Eq. (2) for voltage pulses applied to cell *b* of a pair

1 $${G}_{\text{j}}=\frac{-{I}_{\text{b}}}{{V}_{\text{a}}-{V}_{\text{b}}}$$

2 $${G}_{\text{j}}=\frac{-{I}_{\text{a}}}{{V}_{\text{b}}-{V}_{\text{a}}}$$

where *I*_a_ is *I*_j_ measured in cell *a*, *I*_b_ is *I*_j_ measured in cell *b*, and *V*_a_ and *V*_b_ are the voltages of cell *a* and cell *b*, respectively. Each measurement of *G*_j_ was obtained by plotting *I*_j_ versus the junction voltage (*V*_j_) for a series of different voltage pulses and by calculating *G*_j_ as the slope of a straight line fitted to the *I*_j_–*V*_j_ relationship. For a given cell pair, *G*_j_ was calculated as the average of the *G*_j_ values obtained for both directions of coupling.

For the dual voltage-clamp recordings, the (apparent) membrane resistance was estimated according to Eq. (3) when stepping cell *a* (*r*_m1_)

3 $${r}_{{\text{m}}1}=\frac{{V}_{\text{a}}-{V}_{\text{b}}}{{I}_{\text{a}}+{I}_{\text{b}}}$$

and according to Eq. (4) when stepping cell *b* (*r*_m2_)

4 $${r}_{\text{m}2}=\frac{{V}_{\text{b}}-{V}_{\text{a}}}{{I}_{\text{a}}+{I}_{\text{b}}}$$

We use the term apparent for the membrane resistance estimated from Eqs. (3) and (4) because it only eliminates the contribution from the *G*_j_ between the two cells of a pair, but not that from *G*_j_ between each cell and the other cells to which they are coupled. Each measurement of *r*_m_ was obtained by plotting the voltage versus the current and by calculating *r*_m_ as the slope of a straight line fitted to the *V*–*I* relationship.

In dual recordings of electrically coupled cells, the input resistance (*R*_N_) of either cell cannot be estimated directly when both cells are recorded in voltage clamp. Instead, *R*_N_ was obtained indirectly by calculating it from the apparent membrane resistances (*r*_m1_, *r*_m2_) and *G*_j_ according to Eq. (5) for cell *a* (*R*_N1_) and Eq. (6) for cell *b* (*R*_N2_)

5 $$\frac{1}{{R}_{\text{N}1}}=\frac{1}{{r}_{\text{m}1}}+\frac{1}{{R}_{\text{j}}+{r}_{\text{m}2}} \mathrm\quad {or} \quad{R}_{\text{N}1}=\frac{{r}_{\text{m}1}\left({r}_{\text{m}2}+{R}_{\text{j}}\right)}{{r}_{\text{m}1}+{r}_{\text{m}2}+{R}_{\text{j}}}$$

6 $$\frac{1}{{R}_{\text{N}2}}=\frac{1}{{r}_{\text{m2}}}+\frac{1}{{R}_{\text{j}}+{r}_{\text{m}1}} \mathrm\quad{or}\quad {R}_{\text{N}2}=\frac{{r}_{\text{m}2}\left({r}_{\text{m}1}+{R}_{\text{j}}\right)}{{r}_{\text{m}1}+{r}_{\text{m}2}+{R}_{\text{j}}}$$

where *r*_m1_ is the apparent membrane resistance of cell *a* (estimated from Eq. (3)), *r*_m2_ is the apparent membrane resistance of cell *b* (estimated from Eq. (4)), and *R*_j_ is the inverse of the junctional conductance *G*_j_ (estimated from Eqs. (1) and (2)).

For whole-cell, voltage-clamp recordings of single bipolar cells, *R*_N_ was estimated from the resistive (steady-state) current responses evoked by 20 ms voltage pulses (− 5 or − 10 mV amplitude) by dividing the nominal voltage pulse amplitude by the baseline-subtracted current response amplitude (averaged over a 4 ms interval from 15 to 19 ms after onset of the voltage pulse). Each current response used for this measurement was obtained by averaging 100 individual responses evoked by consecutive voltage pulses (− 5 or − 10 mV). The same current responses were also used for off-line calculation of *I*_hold_ and *R*_s_. *I*_hold_ was calculated by averaging the baseline current over a 4.5 ms interval preceding onset of the voltage pulse. *R*_s_ was estimated by fitting the decay phase during the voltage pulse with a double exponential function and dividing the amplitude of the voltage pulse by the peak current amplitude extrapolated to the onset of the voltage pulse. When examining the effect of MFA on *R*_N_ and *I*_hold_, we averaged the results for seven data points (obtained over a 3 min period), both during the control period and after application of MFA. The average *I*_hold_ prior to application of MFA was − 5.8 ± 10.0 pA (range − 34 to 21 pA). Following exposure to MFA, the average *I*_hold_ was − 7.3 ± 7.2 pA (range − 34 to 4.2 pA) and the average absolute change in *I*_hold_ was 5.4 pA (range 0.03 to 31 pA; *n* = 32 cells).

Data were analyzed off-line with Fitmaster (HEKA Elektronik), IGOR Pro (WaveMetrics), Excel, and GraphPad Prism (GraphPad Software). Experimental data are presented as means ± S.D. (*n* = number of cells). The number of individual traces included in the averaged current traces in the figures are stated for each case. Statistical analyses with comparisons between or within groups were performed using Student’s two-tailed *t* test (paired or unpaired as appropriate) or one-way ANOVA, as indicated in the text. Differences were considered statistically significant at the *P* < 0.05 level. For illustration purposes, most raw data records were either low-pass filtered (digital non-lagging Gaussian filter; − 3 dB at 0.5–1 kHz) or smoothed by a binomial smoothing function (IGOR Pro) to emphasize the kinetics of the response.

## Results

### Properties of Electrical Coupling Between ON-Cone Bipolar and AII Amacrine Cells

**Fig. 1:**
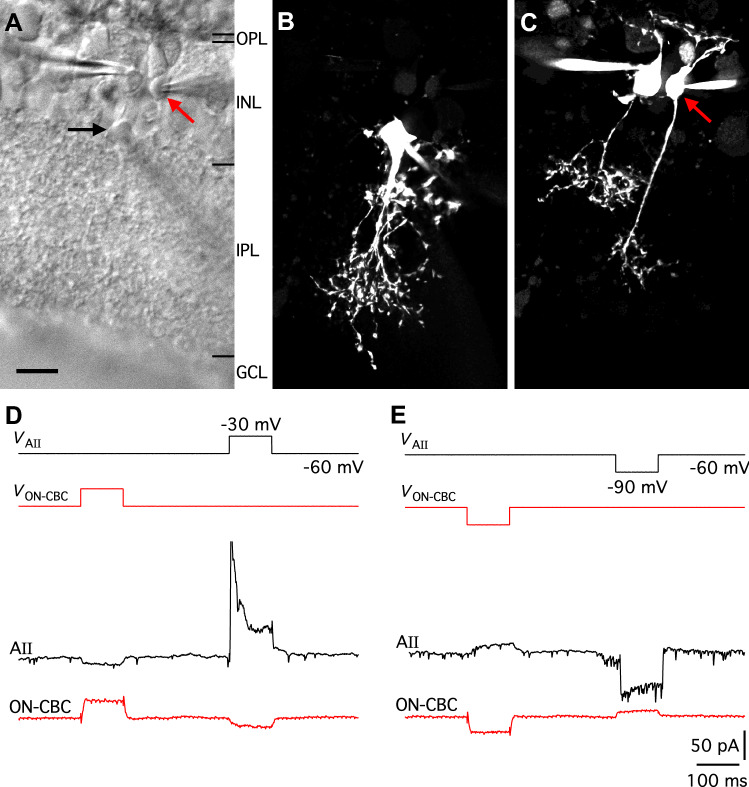
Physiological evidence for gap junction coupling between AII amacrine cells and ON-cone bipolar cells in rat retinal slices. **a** Infrared differential interference contrast (IR-DIC) videomicrograph of a retinal slice with whole-cell recording pipettes attached to an AII amacrine cell (black arrow; cell body visible at border between the inner nuclear layer and the inner plexiform layer) and two cone bipolar cells (cell bodies located in the inner nuclear layer; red arrow points to ON-cone bipolar cell). Same cells in (**a**–**e**). Here and later, retinal layers indicated by black (or white) horizontal lines: *OPL* outer plexiform layer, *INL* inner nuclear layer, *IPL* inner plexiform layer, *GCL* ganglion cell layer. **b** AII amacrine cell filled with Alexa 488 via patch pipette. Here, and in (**c**), maximum intensity projection (MIP; along *Z* axis) generated from wide-field fluorescence stack after deconvolution. **c** OFF-cone bipolar cell (left; type 3) and ON-cone bipolar cell (right, red arrow; type 6) filled with Alexa 594 via patch pipettes. **d**, **e** Simultaneous, dual recording of AII amacrine cell and ON-cone bipolar cell electrically coupled to each other. **d** With both cells in voltage clamp (*V*_hold_ = − 60 mV), 100-ms depolarizing voltage pulses (from *V*_hold_ to − 30 mV) were applied sequentially to AII amacrine cell (*V*_AII_) and ON-cone bipolar cell (*V*_ON-CBC_). Depolarization of AII amacrine cell resulted in outward current in AII (lower black trace) and inward current in ON-cone bipolar cell (lower red trace). Depolarization of ON-cone bipolar cell resulted in outward current in ON-cone bipolar cell (lower red trace) and inward current in AII (lower black trace). **e** With both cells in voltage clamp (*V*_hold_ = − 60 mV), 100-ms hyperpolarizing voltage pulses (from *V*_hold_ to − 90 mV) were applied sequentially to AII amacrine cell (*V*_AII_) and ON-cone bipolar cell (*V*_ON-CBC_). Hyperpolarization of AII amacrine cell resulted in inward current in AII (lower black trace) and outward current in ON-cone bipolar cell (lower red trace). Hyperpolarization of ON-cone bipolar cell resulted in inward current in ON-cone bipolar cell (lower red trace) and outward current in AII (lower black trace). Each trace represents a single trial. For this experiment, no pharmacological blockers were added to the extracellular solution, but for the AII amacrine cell, voltage-gated Na^+^ channels were blocked by QX-314 in the pipette solution. Scale bar: 10 µm (**a**–**c**)

In the mammalian retina, there is strong evidence for electrical coupling between ON-cone bipolar cells and AII amacrine cells (Veruki and Hartveit 2002b; Massey et al. 2003; Trexler et al. 2005). We took advantage of this to examine how MFA influences the membrane properties of cells with verified electrical coupling. The most direct way to demonstrate and quantify functional electrical coupling is by simultaneous, dual recording from visually targeted cells in the in vitro retina. AII amacrine cells can be visually targeted for recording according to the size and location of the cell body in the proximal part of the inner nuclear layer and the thick apical dendrite that tapers as it descends into the inner plexiform layer (Fig. 1a, b). In contrast, ON-cone bipolar cells cannot be directly targeted, but their cell bodies tend to be located distally in the inner nuclear layer, proximal to the majority of rod bipolar cell bodies in the most distal part, close to the outer plexiform layer (Fig. 1a, c). To increase the likelihood of recording from electrically coupled pairs of AII amacrine cells and ON-cone bipolar cells, we first targeted an AII amacrine cell and then searched for a presumed ON-cone bipolar cell body as close as possible to a vertical line across the inner nuclear layer that passed through the AII cell body. All cells were filled with fluorescent dyes and the complete morphologies were visualized during (MPE microscopy) or after (wide-field fluorescence microscopy) the electrophysiological recording.

An example of an AII amacrine cell and an ON-cone bipolar cell that were electrically coupled can be seen in Fig. 1. In this example, a total of three cells (one AII amacrine and two cone bipolar cells) were targeted and recorded (Fig. 1a). Fluorescence imaging of the AII amacrine displayed the typical bistratified morphology with arboreal and lobular dendrites in the proximal and distal parts of the inner plexiform layer, respectively (Fig. 1b). The two cone bipolar cells, filled with a different dye than the AII, were visualized separately (Fig. 1c). The bipolar cells could be identified as specific cell types based on the stratification and shape of their axon terminals within the inner plexiform layer (Euler and Wässle 1995; Hartveit 1997). Of the two bipolar cells, one was a type 6 ON-cone bipolar (Fig. 1c, right), and the other was a type 3 OFF-cone bipolar cell (Fig. 1c, left). The OFF-cone bipolar cell was leaky and only weakly connected through a chemical synapse to the AII, and as it is not relevant to the results presented here, it will not be further commented on.

In such a paired recording of an AII amacrine and an ON-cone bipolar cell, with both cells recorded in whole-cell voltage clamp, electrical coupling is immediately apparent when a voltage pulse is applied to either cell. A depolarizing voltage pulse (+ 30 mV relative to *V*_hold_ = − 60 mV) applied to the ON-cone bipolar cell or the AII amacrine cell (i.e., the presynaptic cell) evoked an inward current in the postsynaptic cell (i.e., the non-stepped cell; Fig. 1d). Correspondingly, a hyperpolarizing voltage pulse (− 30 mV relative to *V*_hold_ = − 60 mV) applied to the ON-cone bipolar cell or the AII amacrine cell evoked an outward current in the postsynaptic cell (Fig. 1e). For each direction of coupling, we estimated *G*_j_ as the slope of a straight line fitted to the *I*_j_ versus *V*_j_ relationship. As previously observed in our laboratory (Veruki and Hartveit 2002b), the junctional conductance was very similar for both directions of coupling and we calculated *G*_j_ as the average of the conductance values measured for each direction. For this cell pair the junctional conductance was calculated to be ~ 400 pS.

### MFA Blocks Electrical Coupling Between ON-Cone Bipolar and AII Amacrine Cells

Using paired recordings from electrically coupled ON-cone bipolar cells and AII amacrine cells, we have previously demonstrated that MFA blocks the electrical synapses between these cells (Veruki and Hartveit 2009). Here, we have re-analyzed data obtained in the previous study to quantify the effect of MFA on input resistance (*R*_N_) and apparent membrane resistance (*r*_m_) and, importantly, to examine how the MFA-evoked changes of these parameters develop in parallel with the gradual reduction of the junctional conductance (*G*_j_). This analysis is similar to that previously reported from our laboratory for pharmacological uncoupling of AII amacrine cells (Veruki et al. 2010). The dual recording paradigm provides direct evidence for the block of electrical coupling by MFA and predicts that the concomitant changes of membrane properties can be expected to occur during similar recording from single cells.

**Fig. 2:**
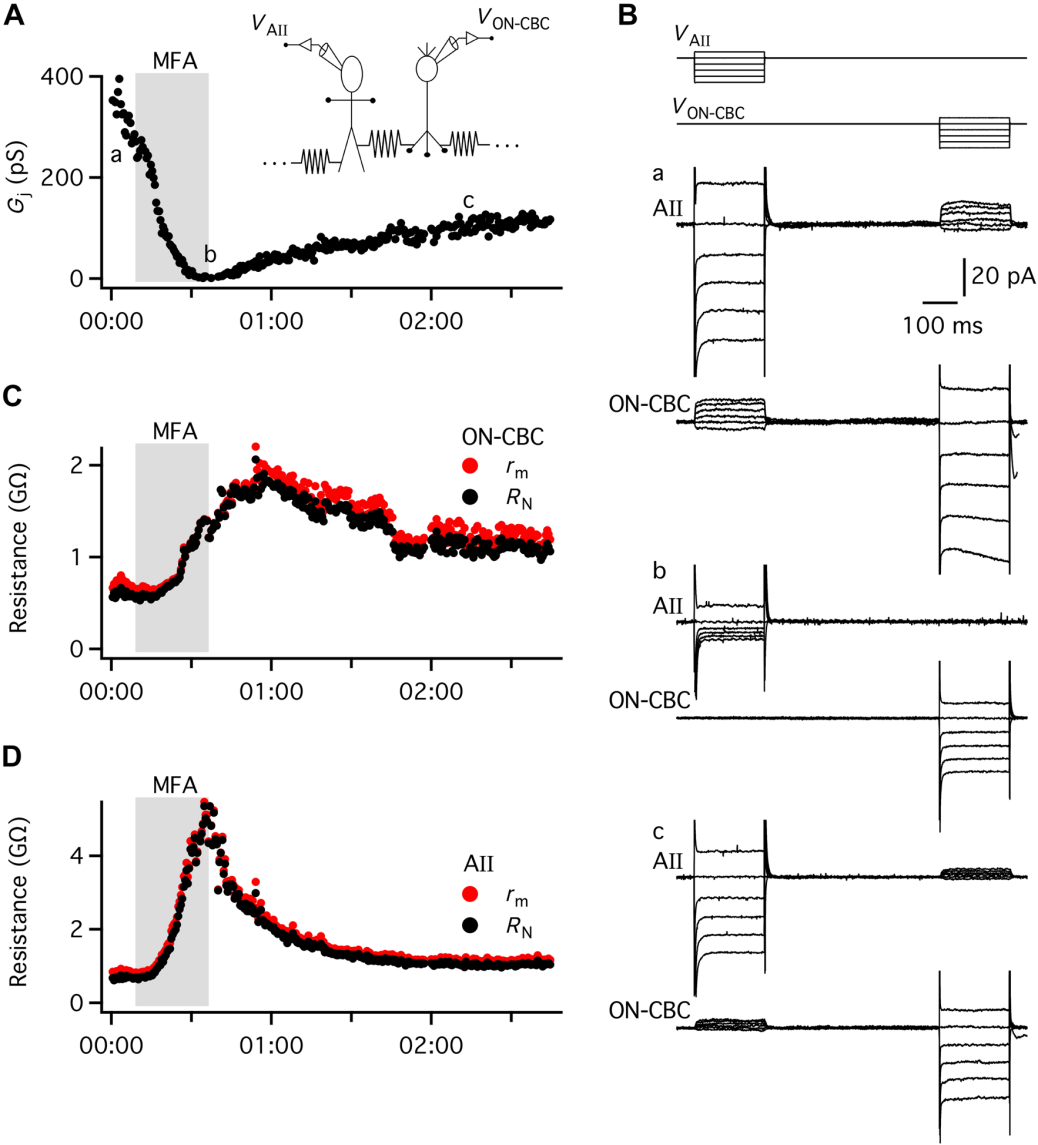
Changes in apparent membrane resistance (apparent *r*_m_) and input resistance (*R*_N_) of ON-cone bipolar and AII amacrine cells accompany block of electrical coupling by meclofenamic acid (MFA). **a** Junctional conductance (*G*_j_) as a function of time during paired recording of an AII amacrine cell and an ON-cone bipolar cell (type 5 or 6) electrically coupled to each other (dual whole-cell voltage-clamp recording with DSEVC amplifiers). *G*_j_ is calculated as the average of the conductance values measured for each direction of coupling (with voltage pulses applied to either ON-cone bipolar or AII amacrine). MFA was applied in the extracellular solution during the period indicated by the shaded area (duration ~ 30 min). The lowercase letters a, b, and c indicate time points during control (a), in the presence of MFA (b), and after washout of MFA (c), where current responses are displayed in (**b**). Same cell pair in (**a**–**d**). *Inset* schematic diagram of recording configuration with resistor between the two recorded cells to indicate electrical coupling and dots extending laterally from resistors attached to each cell to indicate electrical coupling to other AII amacrine and ON-cone bipolar cells. **b** Top: voltage protocol with 200-ms voltage pulses (from − 40 to + 10 mV relative to *V*_hold_ = − 60 mV; increments of 10 mV) applied to the cells (*V*_AII_, *V*_ON-CBC_). Bottom a, b, and c: current responses recorded from AII amacrine cell (AII) and ON-cone bipolar cell (ON-CBC) in response to voltage pulses in the control condition before application of MFA (a), after complete block of *G*_j_ in the presence of MFA (b), and after partial recovery of electrical coupling after washout of MFA (c). Notice that the asymmetry of the voltage pulses relative to *V*_hold_ helps to identify the corresponding current responses in the two cells. Current traces are individual responses. Capacitative current transients have been truncated for clarity. **c**, **d** Apparent *r*_m_ and *R*_N_ for ON-cone bipolar cell (**c**) and AII amacrine cell (**d**) as a function of time in the control condition, during application of MFA, and during washout of MFA. For both cells, *r*_m_ and *R*_N_ increased during application of MFA. Throughout the recording period, ionotropic neurotransmitter receptors (non-NMDA glutamate receptors, GABA_A_ receptors, glycine receptors), and voltage-gated Na^+^ channels were blocked by drugs added to the extracellular solution (see “Materials and Methods” section for details)

In a dual recording of an ON-cone bipolar cell and an AII amacrine cell electrically coupled to each other, adding MFA (100 µM) to the bath solution completely blocked the junctional conductance (Fig. 2a). The onset of block was rapid and could essentially be observed as soon as MFA reached the recording chamber. However, the blocking action of MFA was fairly slow and it typically took ~ 20 to 30 min before the electrical coupling had been completely blocked (Fig. 2a). For the cell pair illustrated in Fig. 2, we were able to maintain the recording for almost 3 h and during washout of MFA, the electrical coupling partially recovered. Figure 2b illustrates example responses of the two cells evoked by the voltage-clamp stimuli at three different time points during the recording: in the control condition (a), during complete block of coupling by MFA (b), and after partial recovery following washout of MFA (c). From a comparison of the responses of each cell in the control condition and during complete block of electrical coupling, it is apparent that the block of coupling by MFA was accompanied by an increase of *R*_N_ for each cell.

To analyze the effect of MFA in more detail, we calculated both *r*_m_ and *R*_N_ for each cell for all measurements during the recording time illustrated in Fig. 2a. In recordings from single cells, all cells electrically coupled to the recorded cell would be free to change their membrane potential. In the paired recording, however, both cells were held in voltage clamp, thus, the postsynaptic cell is not free to change its voltage when the membrane potential of the presynaptic cell is changed. This complicates the calculation of the *R*_N_ of the stepped cell (see “Materials and Methods” section). Figure 2c and d illustrate *r*_m_ and *R*_N_ for the ON-cone bipolar and the AII amacrine cell and how the values changed during application of MFA. In the control condition, *r*_m_ was higher than *R*_N_ (Fig. 2c, d). At the point in time when electrical coupling was completely blocked by MFA, the two values were essentially identical, both for the ON-cone bipolar and the AII amacrine cell. For the ON-cone bipolar cell, *R*_N_ changed from ~ 0.6 GΩ in control to ~ 2 GΩ after complete block of electrical coupling (Fig. 2c). For the AII, the corresponding change was from ~ 0.7 GΩ to ~ 5 GΩ (Fig. 2d). These results suggest that if a retinal neuron is electrically coupled to other neurons, with functional properties of coupling similar to those for ON-cone bipolar cell to AII amacrine cell coupling, it should be possible to detect a change in *R*_N_ when the coupling is blocked. The magnitude of the change will depend both on the total number of cells coupled to the recorded cell and on the junctional conductances between the cells. In paired recordings between ON-cone bipolar cells and AII amacrine cells in rat retina, we have previously measured an average junctional conductance of 1.2  nS (range between 0.1 and 3.3 nS; including types 5, 6, 7, and 8; Veruki and Hartveit 2002b). To our knowledge, however, there is no information concerning the number of ON-cone bipolar cells directly coupled to an AII amacrine cell in rat retina.

### MFA Evokes a Marked Increase of the Input Resistance of ON-Cone Bipolar Cells

**Fig. 3:**
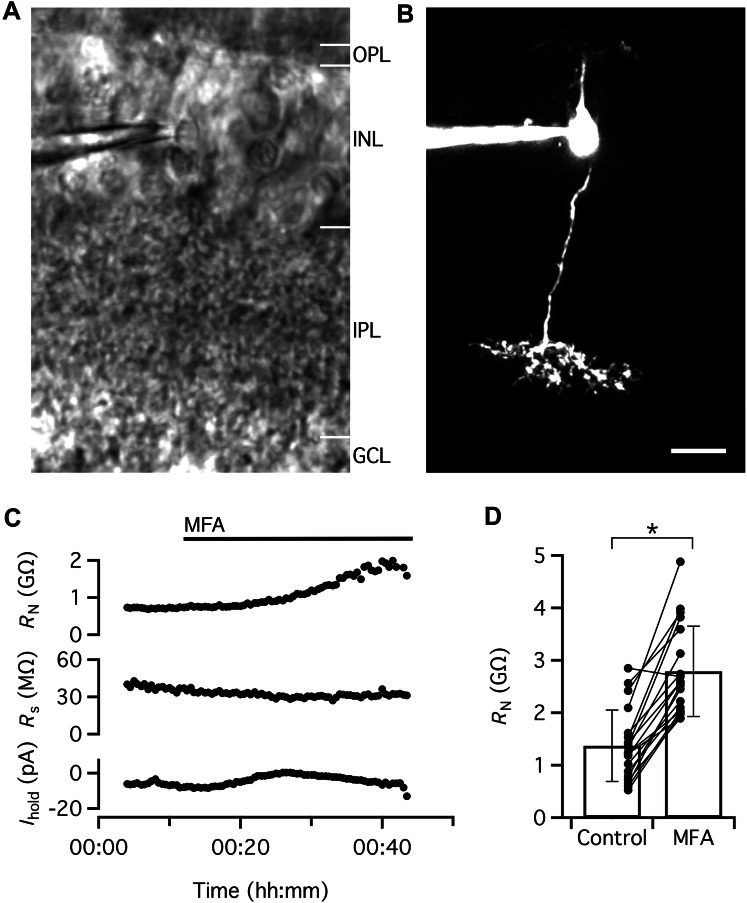
MFA evokes a marked increase of *R*_N_ of ON-cone bipolar cells. **a** Infrared laser scanning gradient contrast (IR-LSGC) image shows recording pipette and retinal slice during whole-cell recording and multiphoton excitation (MPE) microscopy. **b** ON-cone bipolar cell filled with Alexa 594 via patch pipette during whole-cell recording. MIP (along *Z* axis) generated from MPE microscopy fluorescence image stack after deconvolution. Morphological characteristics and level of stratification of axon terminal in inner plexiform layer allow identification of cell as a type 6 ON-cone bipolar cell. Same cell in (**a**–**c**). **c** *R*_N_, *R*_s_, and voltage-clamp holding current (*I*_hold_; *V*_hold_ = − 60 mV) obtained in control condition and after addition of MFA (100 µM) to the extracellular solution to block electrical coupling via gap junctions (period of application indicated by the horizontal line at top). Here and in Figs. 4 and 5, the time series plots of *R*_N_, *R*_s_, and *I*_hold_ were obtained by analyzing the current responses evoked by repeated application of − 5 and − 10 mV voltage pulses (20 ms). Each data point was obtained by analysis of a current waveform obtained by averaging 100 consecutive responses (see “Materials and Methods” section for details on analysis procedures). Throughout the recording period illustrated here (and in Figs. 4 and 5), ionotropic neurotransmitter receptors (non-NMDA and NMDA glutamate receptors, GABA_A_ and GABA_C_ receptors, glycine receptors), voltage-gated Na^+^ channels, and *I*_h_ were blocked by drugs added to the extracellular solution (see “Materials and Methods” section for details). Notice marked increase of *R*_N_ following application of MFA (with smaller changes in *R*_s_ and *I*_hold_). **d** *R*_N_ in the control condition and in the presence of MFA (100 µM) for ON-cone bipolar cells (*n* = 19 cells) investigated as in (**c**). Here and later, bars represent mean ± SD, data points for the same cell are connected by lines, and the results from statistical comparisons between averages are indicated by n.s. (no significant difference; *P* > 0.05) or a single asterisk (statistically significant difference; *P* ≤ 0.05). Notice that the majority of ON-cone bipolar cells display a marked increase of *R*_N_ in the presence of MFA. Scale bar: 10 µm (**a**, **b**)

We next recorded from single ON-cone bipolar cells in voltage clamp (*V*_hold_ = − 60 mV) and measured *R*_N_ by applying small voltage pulses (− 5 and − 10 mV) relative to *V*_hold_. Throughout the recording period, the bath solution contained pharmacological blockers of neurotransmitter receptors (glutamate, GABA, glycine), voltage-gated Na^+^ channels and *I*_h_ (see “Materials and Methods” section). For the cell illustrated in Fig. 3a–c (a type 6 cone bipolar cell), *R*_N_ in the control condition was ~ 0.74 GΩ. After ~ 12 min recording, we added MFA to the bath and continued the recording for another ~ 30 min (Fig. 3c). During this period, there was a gradual increase of *R*_N_, with a maximum of ~ 2.0 GΩ (Fig. 3c). There was a slight reduction of *R*_s_ during the recording period, but no consistent change in *I*_hold_.

Similar results were observed for a total of 19 ON-cone bipolar cells (Fig. 3d). Of these cells, nine were type 5, four were type 6, four were type 7, and two were type 8. For all but one cell (a type 6), application of MFA evoked a marked increase of *R*_N_ relative to the value obtained for the control condition (with pharmacological blockers). In control, the average value of *R*_N_ was 1.37 ± 0.68 GΩ (range 0.52–2.85 GΩ) and in MFA, the average value of *R*_N_ was 2.79 ± 0.86 GΩ (range 1.89–4.88 GΩ; *P* = 3.8 × 10^–7^, paired *t* test; *n* = 19 cells; Fig. 3d).

### MFA Does Not Increase the Input Resistance of OFF-Cone Bipolar Cells

**Fig. 4:**
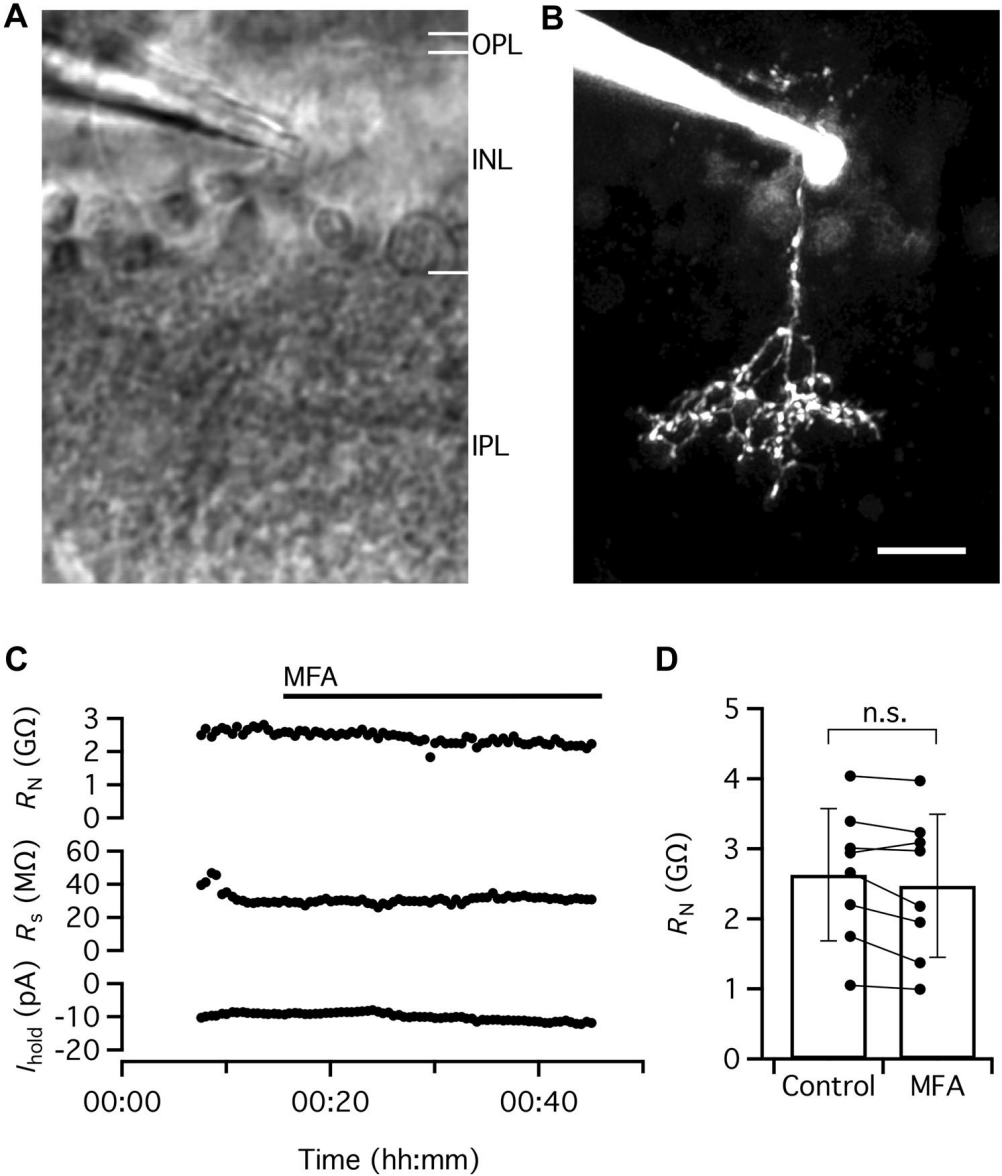
MFA does not increase *R*_N_ of OFF-cone bipolar cells. **a** IR-LSGC image shows recording pipette and retinal slice during whole-cell recording and MPE microscopy. **b** OFF-cone bipolar cell filled with Alexa 594 via patch pipette during whole-cell recording. MIP (along *Z* axis) generated from MPE microscopy fluorescence image stack after deconvolution. Morphological characteristics and level of stratification of axon terminal in inner plexiform layer allow identification of cell as a type 3 OFF-cone bipolar cell. Same cell in (**a**–**c**). **c** *R*_N_, *R*_s_ and voltage-clamp *I*_hold_ (*V*_hold_ = − 60 mV) obtained in control condition and after addition of MFA (100 µM) to the extracellular solution (period of application indicated by the horizontal line at top). Notice small decrease of *R*_N_ following application of MFA (with little change in *R*_s_ and *I*_hold_). **d** *R*_N_ in the control condition and in the presence of MFA (100 µM) for OFF-cone bipolar cells (*n* = 8 cells) investigated as in (**c**). Scale bar: 10 µm (**a**, **b**)

Similar to the experiments with ON-cone bipolar cells, we also recorded from single OFF-cone bipolar cells in voltage clamp (*V*_hold_ = − 60 mV) and repeatedly measured *R*_N_ by applying small voltage pulses (− 5 and − 10 mV) relative to *V*_hold_. For the cell illustrated in Fig. 4a–c (a type 3 cone bipolar cell), *R*_N_ in the control condition was ~ 2.5 GΩ. For comparison, we have also illustrated *R*_s_ and *I*_hold_ in the same graph (Fig. 4c). After 15 min of recording, we added MFA to the bath and continued recording for another 30 min (Fig. 4c). During this period, there was only a small, gradual reduction of *R*_N_ to ~ 2.2 GΩ by the end of the recording period (Fig. 4c). There were only minimal changes in the values for *R*_s_ and *I*_hold_ (Fig. 4c).

Similar results were observed for a total of eight OFF-cone bipolar cells (Fig. 4d). Of these cells, two were type 2, three were type 3, and three were type 4. In the control condition (with pharmacological blockers), the average value of *R*_N_ was 2.63 ± 0.95 GΩ (range 1.05–4.04 GΩ) and after applying MFA the average value of *R*_N_ was 2.47 ± 1.02 GΩ (range 0.99–3.97 GΩ; *P* = 0.0605, paired *t* test; *n* = 8 cells; Fig. 4d). It is possible that the small reduction of *R*_N_ observed in the presence of MFA for seven of the cells might be caused by an effect of MFA on ion channels other than gap junction channels. For example, MFA has been shown to inhibit hKv2.1 potassium channels (Lee and Wang 1999), open KCNQ2/Q3 potassium channels (Peretz et al. 2005) and stimulate BK channel activity (Wu et al. 2001).

### MFA Does Not Increase the Input Resistance of Rod Bipolar Cells

**Fig. 5:**
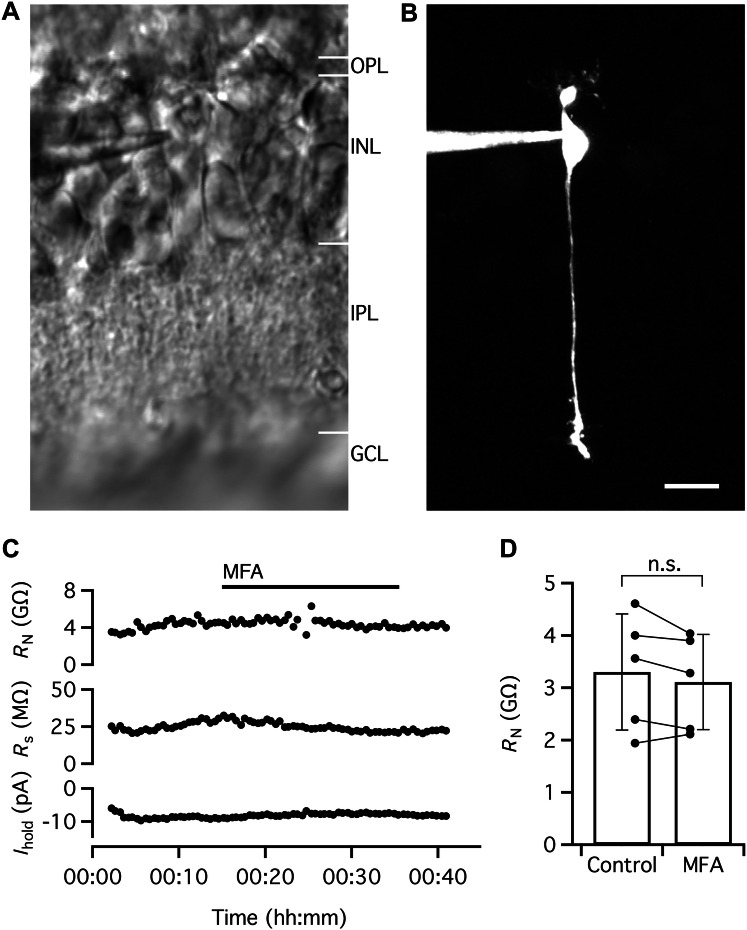
MFA does not increase *R*_N_ of rod bipolar cells. **a** IR-LSGC image shows recording pipette and retinal slice during whole-cell recording and MPE microscopy. **b** Rod bipolar cell filled with Alexa 594 via patch pipette during whole-cell recording. MIP (along *Z* axis) generated from MPE microscopy fluorescence image stack after deconvolution. Morphological characteristics and level of stratification of axon terminal in inner plexiform layer allow identification of cell as a rod bipolar cell. Same cell in (**a**–**c**). **c** *R*_N_, *R*_s_, and voltage-clamp *I*_hold_ (*V*_hold_ = − 60 mV) obtained in control condition and after addition of MFA (100 µM) to the extracellular solution (period of application indicated by the horizontal line at top). Notice that there is no consistent change of *R*_N_ following application of MFA (with smaller changes in *R*_s_ and *I*_hold_). **d** *R*_N_ in the control condition and in the presence of MFA (100 µM) for rod bipolar cells (*n* = 5 cells) investigated as in (**c**). Scale bar: 10 µm (**a**, **b**)

Gap junctions between rod bipolar cells have not been reported (e.g., Strettoi et al. 1990). Consistent with this, in a previous study from our laboratory, we never observed any evidence for electrical coupling during paired recordings between neighboring rod bipolar cells (Veruki et al. 2006). To examine the effect of MFA, we recorded from single rod bipolar cells in voltage clamp (*V*_hold_ = − 60 mV) and measured *R*_N_ by applying small voltage pulses (− 5 and − 10 mV) relative to *V*_hold_. For the cell illustrated in Fig. 5a–c, the input resistance in the control condition was ~ 4.0 GΩ. After 15 min of recording, we added MFA to the bath and continued the recording for another ~ 33 min (Fig. 5c). During this period, *R*_N_ was relatively stable, with minor fluctuations around 4 GΩ during the period of ~ 20 min when MFA was applied (Fig. 5c). There were only moderate changes of *R*_s_ and *I*_hold_ during the recording period. Similar results were observed for a total of five rod bipolar cells (Fig. 5d). In control, the average value of *R*_N_ was 3.3 ± 1.1 GΩ (range 1.9–4.6 GΩ) and in MFA, the average value of *R*_N_ was 3.1 ± 0.9 GΩ (range 2.1–4.0 GΩ; *P* = 0.1847, paired *t* test; *n* = 5 cells; Fig. 5d).

### Comparisons Within ON- and OFF-Cone Bipolar Cells Reveal No Type-Specific Effects of MFA

**Fig. 6:**
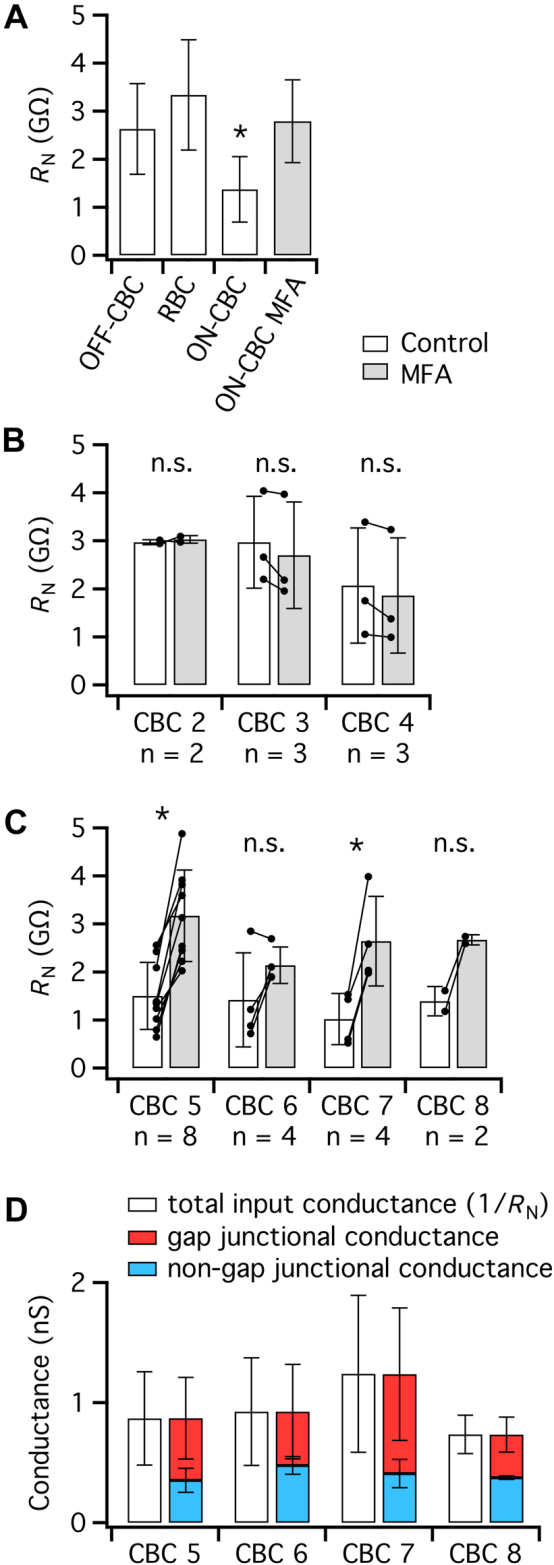
Overview of *R*_N_ and non-gap junctional versus gap junctional conductance for different types of OFF- and ON-cone bipolar cells. **a** Comparison of *R*_N_ for OFF-cone, rod, and ON-cone bipolar cells in control condition and ON-cone bipolar cells in the presence of MFA (100 µM) to block gap junctions. Notice that *R*_N_ of ON-cone bipolar cells in control is significantly different from the other three groups, which are not significantly different from each other (for statistics, see “Results” section). Here and in **b **and **c**, open bars indicate control and gray bars indicate MFA. **b** *R*_N_ in control condition and in MFA for three different types of OFF-cone bipolar cells. **c** *R*_N_ in the control condition and in the presence of MFA for four different types of ON-cone bipolar cells. Notice that except for one type 6 cell, all types of ON-cone bipolar cells display a marked increase of *R*_N_ in the presence of MFA. **d** Total input conductance (= 1/*R*_N_) and the relation between gap junctional conductance and non-gap junctional conductance for four different types of ON-cone bipolar cells. To illustrate the variability of the total input conductance and its two components, each cell type is represented with two bars. There was no statistically significant difference between either the gap junctional conductance or the non-gap junctional conductance for the four cell types (see “Results” section)

The results so far presented reveal that MFA evoked a marked increase of *R*_N_ for ON-cone bipolar cells, but not for OFF-cone and rod bipolar cells. When we compared the average values of *R*_N_ for the different cell types in the control condition, we observed little difference between *R*_N_ for OFF-cone and rod bipolar cells, but both values were higher than *R*_N_ for ON-cone bipolar cells (Fig. 6a). Notably, the average *R*_N_ for ON-cone bipolar cells in MFA was very similar to *R*_N_ for both OFF-cone and rod bipolar cells in control (Fig. 6a). A statistical comparison of the values of *R*_N_ for the four groups illustrated in Fig. 6a, confirmed that there was no difference between *R*_N_ for ON-cone bipolars in MFA, OFF-cone bipolars in control, and rod bipolars in control, whereas *R*_N_ for ON-cone bipolars in control was significantly smaller than *R*_N_ for the other three groups (*P* ≥ 0.5055 and *P* ≤ 0.0044, respectively, *F*_(3,47)_ = 0.6581; one-way ANOVA, Tukey’s multiple comparison test, adjusted *P* values). This suggests that with respect to *R*_N_, closing gap junction channels makes ON-cone bipolar cells electrically similar to OFF-cone and rod bipolar cells.

We next asked whether there was any evidence for type-specific effects of MFA on *R*_N_ that were potentially masked in our previous analysis where all types among ON- and OFF-cone bipolar cells were grouped together. When we compared the different types of ON- and OFF-cone bipolar cells (cells in Figs. 3d and 4d, respectively,) in the control condition, we found no statistically significant difference between the values of *R*_N_ for the different types of ON-cone bipolar cells (*F*_(3,15)_ = 0.4214, *P* = 0.7403, one-way ANOVA) or for the different types of OFF-cone bipolar cells (*F*_(2,5)_ = 0.8162, *P* = 0.4935, one-way ANOVA). Accordingly, the relatively large variability of *R*_N_ in the control condition for both OFF- and ON-cone bipolar cells (Figs. 3d and 4d) cannot be explained by systematic differences between the different types of bipolar cells within each group.

We next compared the effect of MFA on the different types of OFF- and ON-cone bipolar cells (Fig. 6b, c). Although the group sizes are small, MFA had no statistically significant effect on *R*_N_ for any of the three types of OFF-cone bipolar cells (Fig. 6b). For ON-cone bipolar cells, MFA had the same qualitative effect on all types of cells, with clear increases in *R*_N_ (Fig. 6c). Here, the increase in *R*_N_ was statistically significant for cell types 5 and 7, but not for type 8 (where we only had two cells) and type 6 (where one of the cells showed a decrease). We were unfortunately not successful in obtaining recordings for type 1 (OFF) and type 9 (ON) cone bipolar cells, both of which are relatively rare types (Euler et al. 2014). Taken together, we can summarize these results by concluding that we find no evidence for a type-specific effect of MFA within either ON- or OFF-cone bipolar cells.

Finally, we compared the relative magnitude of the gap junctional conductance and the non-gap junctional conductance for the different types of ON-cone bipolar cells (Fig. 6D). The non-gap junctional conductance was estimated as the input conductance (1/*R*_N_) after blocking gap junctions in the presence of MFA. The gap junctional conductance was calculated by subtracting the non-gap junctional conductance from the total input conductance measured in the control condition. There was no statistically significant difference between either the gap junctional conductance (*F*_(3,15)_ = 1.008, *P* = 0.4166, one-way ANOVA) or the non-gap junctional conductance for the four cell types (*F*_(3,15)_ = 1.911, *P* = 0.1712,, one-way ANOVA). This suggests that for the four types of ON-cone bipolar cells recorded from here, the contributions of gap junctional and non-gap junctional conductance to the total membrane conductance are similar.

## Discussion

The function of morphologically identified gap junctions between cone bipolar cells is puzzling. As a first step towards elucidating potential roles for these gap junctions, we investigated whether their presence between bipolar cells makes a measurable contribution to the total input resistance of these cells. We measured *R*_N_ of single bipolar cells in a rat retinal slice preparation under control conditions and in the presence of MFA, a well-documented pharmacological blocker of gap junctions (Pan et al. 2007; Veruki and Hartveit 2009). For ON-cone bipolar cells, we observed a marked increase of *R*_N_ in MFA. For OFF-cone bipolar cells, however, MFA did not increase *R*_N_ and these cells appeared similar to rod bipolar cells, for which there is no morphological evidence for gap junctions. If we accept the existing evidence for the presence of gap junctions between cone bipolar cells, the simplest interpretation of our results is that for OFF-cone bipolar cells, the open probability of the corresponding gap junction channels (connexons) is so low that blocking the channels has no detectable influence on the electrotonic properties of the cells. In the following, we discuss our results in the context of the evidence for gap junctions between cone bipolar cells.

### Evidence for Gap Junctions Involving Cone Bipolar Cells

For OFF-cone bipolar cells, there are a number of studies in a variety of species that strongly suggest the presence of gap junctions, both between the dendrites (Mills 1999; Feigenspan et al. 2004; O'Brien et al. 2012; Kántor et al. 2016) and the axon terminals (Kolb 1979; Jacoby and Marshak 2000; Tsukamoto and Omi 2015, 2017; Kántor et al. 2017) of these cells. In addition, there is evidence that the gap junctions at the dendrites of OFF-cone bipolar cells contain Cx36 (Feigenspan et al. 2004; O'Brien et al. 2012; Kántor et al. 2016). Heterologous gap junctions between adjacent ON-cone bipolars have also been reported (Cohen and Sterling 1990; Tsukamoto and Omi 2017). In mouse retina, it has been reported that the gap junctions between OFF-cone bipolar cells were more frequently encountered than those between ON-cone bipolar cells (Tsukamoto and Omi 2017). The majority of gap junction connections involving ON-cone bipolar cells are with AII amacrine cells (Kolb and Famiglietti 1974; McGuire et al. 1984; Strettoi et al. 1992, 1994; Chun et al. 1993). In addition, there is direct evidence from dual recordings of pairs of ON-cone bipolar and AII amacrine cells that these gap junctions function as electrical synapses (Veruki and Hartveit 2002b, 2009). There is also morphological evidence that the axon terminals of ON-cone bipolar cells make gap junctions with the bistratified, narrow-field A8 amacrine cell (Kolb and Nelson 1996; Lee et al. 2015), but as far as we know, direct evidence for functional coupling between these cell types is lacking.

### Why Does MFA Not Increase the Input Resistance of OFF-Cone Bipolar Cells?

Given the strong evidence for gap junctions located at both the dendrites and axon terminals of OFF-cone bipolar cells, it is surprising that MFA did not increase *R*_N_ of these cells. Such an increase would be expected if MFA blocked open gap junction channels that contributed to the input conductance of the cells, similar to the results observed here for ON-cone bipolar cells and the results for AII amacrine cells, A17 amacrine cells, and Golgi cell interneurons, as discussed earlier. There are several possible explanations for the lack of effect of MFA which will be discussed below.

First, the possibility has to be considered whether MFA is unable to block the channels (connexons) of OFF-cone bipolar gap junctions. For several reasons, we consider this possibility unlikely. In rabbit retina, MFA blocks dye/tracer coupling of both A-type horizontal cells, B-type horizontal cells and AII amacrine cells (Pan et al. 2007), all of which are thought to express different connexins (Cx50, Cx57 and Cx36, respectively). In rat retina, MFA blocks electrical coupling between pairs of AII amacrine cells, pairs of ON-cone bipolar cells and AII amacrine cells, and pairs of A17 amacrine cells (Veruki and Hartveit 2009; Veruki et al. 2010; Elgueta et al. 2018; Zandt et al. 2018). There is evidence that gap junctions between ON-cone bipolar cells and AII amacrine cells are either homomeric, with both neurons expressing Cx36, or heteromeric with the AII expressing Cx36 and the bipolar cell expressing Cx45 (Maxeiner et al. 2005; Han and Massey 2005; Lin et al. 2005; Dedek et al. 2006). Taken together, these results suggest that there is little selectivity between different connexins for block by MFA. A similar lack of selectivity was also found for the potent block of Cx36 and Cx50 by mefloquine (Cruikshank et al. 2004). Although it is hard to exclude the possibility that OFF-cone bipolar cell gap junctions contain a connexin with little sensitivity to MFA, the existing evidence suggests the presence of Cx36 and/or Cx45 in bipolar cells (Feigenspan et al. 2004; Han and Massey 2005; Maxeiner et al. 2005; Lin et al. 2005**)** which should be blocked by MFA (Pan et al. 2007; Veruki and Hartveit 2009). In addition, the nearly identical values obtained for the *R*_N_ of ON-cone bipolar cells in MFA and OFF-cone bipolar cells in the control condition suggest that the lack of effect of MFA on OFF-cone bipolar cells is due to the absence of electrical coupling (open gap junction channels) between these cells, rather than the presence of MFA-resistant electrical coupling. This observation suggests that OFF-cone bipolar cells in the rat retina are effectively uncoupled electrically.

Second, it seems difficult to escape the conclusion that the gap junction channels between OFF-cone bipolar cells, irrespective of whether they are located at dendrites or axon terminals (or both), do not contribute a measurable conductance under our recording conditions. The total conductance contributed by gap junction channels is a function of the number of channels, the single-channel conductance, and the (average) open probability. To our knowledge, there are no estimates of the total number of connexons associated with OFF-cone bipolar cells, although there are reports that the gap junctions are relatively small (Kolb 1979). It is also possible that the open probability could be very low. Because of the electrical coupling between ON-cone bipolar cells and AII amacrine cells, the present results do not permit strong conclusions about the functional properties of the gap junctions directly between ON-cone bipolar cells. Currently, it is not possible to differentiate experimentally between the relative contribution of heterologous and (potential) homologous electrical synapses of ON-cone bipolar cells. However, it is a possibility that the gap junctions between ON-cone bipolar cells, analogous to those between OFF-cone bipolar cells, contribute little measurable conductance to these cells.

### A Functional Role for Electrical Coupling Between Cone Bipolar Cells?

If we assume that at least under some conditions, the strength of gap junction coupling between OFF-cone bipolar cells and, potentially, directly between ON-cone bipolar cells is sufficiently strong to mediate measurable electrical coupling, this raises the question of a functional role. To our knowledge, the only bipolar cells for which electrical coupling has been unequivocally demonstrated are the Mb1 (ON) bipolar cells in goldfish retina (Arai et al. 2010). These cells are electrically coupled to each other at their distal dendrites. With dual recording of electrically coupled cell pairs, it was demonstrated that the electrical coupling acted as a low-pass filter that could transmit Ca^2+^ spikes, leading to a prolongation of postsynaptic currents in ganglion cells, and it was suggested that the electrical coupling might play a role in lateral interactions (Arai et al. 2010). Interestingly, it was also reported that the strength of coupling was larger under light-adapted conditions.

It has been suggested that although electrical coupling between OFF-cone bipolar cells would reduce spatial acuity, it could contribute to an improved signal-to-noise ratio (Mills 1999; Feigenspan et al. 2004). There does not seem to be experimental data to verify or refute this idea, but unless this mechanism could work at very low levels of coupling, it does not seem consistent with the results for OFF-cone bipolar cells obtained in our study.

Recently it has been shown that electrical synapses at the axons of ON-cone bipolar cells in mouse retina contribute to a lateral spread of visual signals that leads to increased sensitivity in retinal ganglion cells to spatiotemporally correlated inputs such as motion (Kuo et al. 2016). This lateral spread was postulated to be mediated via electrical synapses between AII amacrine cells and ON-cone bipolar cells, but additional contributions from direct coupling between ON-cone bipolar cells could not be ruled out (Kuo et al. 2016). A similar lateral spread could be mediated by gap junctions between OFF-cone bipolar cell axon terminals, but as discussed above, it is unclear whether such lateral spread could be mediated by the very low levels of coupling consistent with our results.

In general, there is strong evidence that the open probability of different connexons is under modulatory control (for review, see O'Brien 2019), including connexons expressed in retinal neurons (for review, see Trenholm and Awatramani 2017; O'Brien and Bloomfield 2018). Currently, nothing is known concerning potential modulation of the strength of gap junction coupling between OFF-cone bipolar cells in the mammalian retina, but it is possible that the strength of coupling could be stronger under conditions not explored in our recordings. However, if the strength of coupling between OFF-cone bipolar cells (and, by analogy, between ON-cone bipolar cells) is permanently too weak to impart functionally relevant electrical coupling, the main purpose of the gap junction channels could be to enable chemical and/or metabolic coupling within local subcellular domains, or to play a structural role, e.g., as adhesion molecules (cf. Mills 1999; Pereda 2016).

### Future Perspectives

It will be experimentally challenging to systematically investigate potential electrical coupling between cone bipolar cells by attempting to perform simultaneous dual patch-clamp recording from pairs of coupled cells. Without access to animals with fluorescently labeled cell types, the success rate of targeting two specific OFF-cone bipolar cells or two specific ON-cone bipolar cells is likely to be discouragingly low, given the number of different types of bipolar cells in mammalian retina, including rat (Euler and Wässle 1995; Hartveit 1997) and mouse (Ghosh et al. 2004; Helmstaedter et al. 2013; Euler et al. 2014) which are the experimentally most relevant species. In addition, the findings from our study suggest that the strength of coupling is likely to be very low. If the coupling strength is physiologically regulated, a favorable strategy might be to first identify the condition(s) under which the junctional conductance is upregulated.

## References

[CR1] Alcamí P, Pereda AE (2019) Beyond plasticity: the dynamic impact of electrical synapses on neural circuits. Nat Rev Neurosci 20:253–271 30824857 10.1038/s41583-019-0133-5

[CR2] Arai I, Tanaka M, Tachibana M (2010) Active roles of electrically coupled bipolar cell network in the adult retina. J Neurosci 30:9260–9270 20610761 10.1523/JNEUROSCI.1590-10.2010 PMC6632464

[CR3] Bloomfield SA, Dacheux RF (2001) Rod vision: pathways and processing in the mammalian retina. Prog Retin Eye Res 20:351–384 11286897 10.1016/s1350-9462(00)00031-8

[CR4] Castilho Á, Ambrósio AF, Hartveit E, Veruki ML (2015) Disruption of a neural microcircuit in the rod pathway of the mammalian retina by diabetes mellitus. J Neurosci 35:5422–5433 25834065 10.1523/JNEUROSCI.5285-14.2015 PMC6705407

[CR5] Chun MH, Han SH, Chung JW, Wässle H (1993) Electron microscopic analysis of the rod pathway of the rat retina. J Comp Neurol 332:421–432 8349841 10.1002/cne.903320404

[CR6] Cohen E, Sterling P (1990) Demonstration of cell types among cone bipolar neurons of cat retina. Philos Trans R Soc Lond B Biol Sci 330:305–321 1982357 10.1098/rstb.1990.0201

[CR7] Cruikshank SJ, Hopperstad M, Younger M, Connors BW, Spray DC, Srinivas M (2004) Potent block of Cx36 and Cx50 gap junction channels by mefloquine. Proc Natl Acad Sci USA 101:12364–12369 15297615 10.1073/pnas.0402044101 PMC514481

[CR8] Dedek K, Schultz K, Pieper M, Dirks P, Maxeiner S, Willecke K, Weiler R, Janssen-Bienhold U (2006) Localization of heterotypic gap junctions composed of connexin45 and connexin36 in the rod pathway of the mouse retina. Eur J Neurosci 24:1675–1686 17004931 10.1111/j.1460-9568.2006.05052.x

[CR9] DeVries SH, Qi X, Smith R, Makous W, Sterling P (2002) Electrical coupling between mammalian cones. Curr Biol 12:1900–1907 12445382 10.1016/s0960-9822(02)01261-7

[CR10] Elgueta C, Leroy F, Vielma AH, Schmachtenberg O, Palacios AG (2018) Electrical coupling between A17 cells enhances reciprocal inhibitory feedback to rod bipolar cells. Sci Rep 8:3123 29449585 10.1038/s41598-018-21119-0 PMC5814567

[CR11] Euler T, Wässle H (1995) Immunocytochemical identification of cone bipolar cells in the rat retina. J Comp Neurol 361:461–478 8550893 10.1002/cne.903610310

[CR12] Euler T, Haverkamp S, Schubert T, Baden T (2014) Retinal bipolar cells: elementary building blocks of vision. Nat Rev Neurosci 8:507–519 10.1038/nrn3783 25158357

[CR13] Feigenspan A, Janssen-Bienhold U, Hormuzdi S, Monyer H, Degen J, Söhl G, Willecke K, Ammermüller J, Weiler R (2004) Expression of connexin36 in cone pedicles and OFF-cone bipolar cells of the mouse retina. J Neurosci 24:3325–3334 15056712 10.1523/JNEUROSCI.5598-03.2004 PMC6730041

[CR14] Ghosh K, Bujan S, Haverkamp S, Feigenspan A, Wässle H (2004) Types of bipolar cells in the mouse retina. J Comp Neurol 469:70–82 14689473 10.1002/cne.10985

[CR15] Grimes WN, Hoon M, Briggman KL, Wong RO, Rieke F (2014) Cross-synaptic synchrony and transmission of signal and noise across the mouse retina. eLife 3:e03892 25180102 10.7554/eLife.03892 PMC4174577

[CR16] Han Y, Massey SC (2005) Electrical synapses in retinal ON cone bipolar cells: subtype-specific expression of connexins. Proc Natl Acad Sci U S A 102:13313–13318 16150718 10.1073/pnas.0505067102 PMC1201596

[CR17] Hartveit E (1996) Membrane currents evoked by ionotropic glutamate receptor agonists in rod bipolar cells in the rat retinal slice preparation. J Neurophysiol 76:401–422 8836233 10.1152/jn.1996.76.1.401

[CR18] Hartveit E (1997) Functional organization of cone bipolar cells in the rat retina. J Neurophysiol 77:1716–1730 9114231 10.1152/jn.1997.77.4.1716

[CR19] Hartveit E, Veruki ML (2010) Accurate measurement of junctional conductance between electrically coupled cells with dual whole-cell voltage-clamp under conditions of high series resistance. J Neurosci Methods 187:13–25 20074587 10.1016/j.jneumeth.2009.12.003

[CR20] Helmstaedter M, Briggman KL, Turaga SC, Jain V, Seung HS, Denk W (2013) Connectomic reconstruction of the inner plexiform layer in the mouse retina. Nature 500:168–174 23925239 10.1038/nature12346

[CR21] Hilgen G, von Maltzahn J, Willecke K, Weiler R, Dedek K (2011) Subcellular distribution of connexin45 in OFF bipolar cells of the mouse retina. J Comp Neurol 519:433–450 21192077 10.1002/cne.22526

[CR22] Jacoby RA, Marshak DW (2000) Synaptic connections of DB3 diffuse bipolar cell axons in macaque retina. J Comp Neurol 4161:9–29 10.1002/(sici)1096-9861(20000103)416:1<19::aid-cne3>3.0.co;2-h PMC3341735 10578100

[CR23] Kántor O, Benkő Z, Énzsöly A, Dávid C, Naumann A, Nitschke R, Szabó A, Pálfi E, Orbán J, Nyitrai M, Németh J, Szél Á, Lukáts Á, Völgyi B (2016) Characterization of connexin36 gap junctions in the human outer retina. Brain Struct Funct 221:2963–2984 26173976 10.1007/s00429-015-1082-z

[CR24] Kántor O, Varga A, Nitschke R, Naumann A, Énzsöly A, Lukáts Á, Szabó A, Németh J, Völgyi B (2017) Bipolar cell gap junctions serve major signaling pathways in the human retina. Brain Struct Funct 222:2603–2624 28070649 10.1007/s00429-016-1360-4

[CR25] Kolb H (1979) The inner plexiform layer in the retina of the cat: electron microscopic observations. J Neurocytol 8:295–329 490185 10.1007/BF01236124

[CR26] Kolb H, Famiglietti EV (1974) Rod and cone pathways in the inner plexiform layer of cat retina. Science 186:47–49 4417736 10.1126/science.186.4158.47

[CR27] Kolb H, Nelson R (1996) Hyperpolarizing, small-field, amacrine cells in cone pathways of cat retina. J Comp Neurol 371:415–436 8842896 10.1002/(SICI)1096-9861(19960729)371:3<415::AID-CNE5>3.0.CO;2-5

[CR28] Kujiraoka T, Saito T (1986) Electrical coupling between bipolar cells in carp retina. Proc Natl Acad Sci USA 83:4063–4066 16593707 10.1073/pnas.83.11.4063 PMC323666

[CR29] Kuo SP, Schwartz GW, Rieke F (2016) Nonlinear spatiotemporal integration by electrical and chemical synapses in the retina. Neuron 90:320–332 27068789 10.1016/j.neuron.2016.03.012 PMC4840068

[CR30] Lee YT, Wang Q (1999) Inhibition of hKv2.1, a major human neuronal voltage-gated K^+^ channel, by meclofenamic acid. Eur J Pharmacol 378:349–356 10493112 10.1016/s0014-2999(99)00485-9

[CR31] Lee SC, Meyer A, Schubert T, Hüser L, Dedek K, Haverkamp S (2015) Morphology and connectivity of the small bistratified A8 amacrine cell in the mouse retina. J Comp Neurol 523:1529–1547 25630271 10.1002/cne.23752 PMC4439304

[CR32] Li W, Zhang J, Massey SC (2002) Coupling pattern of S1 and S2 amacrine cells in the rabbit retina. Vis Neurosci 19:119–131 12385625 10.1017/s0952523802191115

[CR33] Li PH, Verweij J, Long JH, Schnapf JL (2012) Gap-junctional coupling of mammalian rod photoreceptors and its effect on visual detection. J Neurosci 32:3552–3562 22399777 10.1523/JNEUROSCI.2144-11.2012 PMC3319459

[CR34] Lin B, Jakobs TC, Masland RH (2005) Different functional types of bipolar cells use different gap-junctional proteins. J Neurosci 25:6696–6701 16014731 10.1523/JNEUROSCI.1894-05.2005 PMC6725416

[CR35] Manookin MB, Beaudoin DL, Ernst ZR, Flagel LJ, Demb JB (2008) Disinhibition combines with excitation to extend the operating range of the OFF visual pathway in daylight. J Neurosci 28:4136–4150 18417693 10.1523/JNEUROSCI.4274-07.2008 PMC2557439

[CR36] Marc RE, Liu WL, Muller JF (1988) Gap junctions in the inner plexiform layer of the goldfish retina. Vision Res 28:9–24 3414003

[CR37] Massey SC, O'Brien JJ, Trexler EB, Li W, Keung JW, Mills SL, O'Brien J (2003) Multiple neuronal connexins in the mammalian retina. Cell Commun Adhes 10:425–430 14681052 10.1080/cac.10.4-6.425.430

[CR38] Maxeiner S, Dedek K, Janssen-Bienhold U, Ammermüller J, Brune H, Kirsch T, Pieper M, Degen J, Krüger O, Willecke K, Weiler R (2005) Deletion of connexin45 in mouse retinal neurons disrupts the rod/cone signaling pathway between AII amacrine and ON cone bipolar cells and leads to impaired visual transmission. J Neurosci 25:566–576 15659592 10.1523/JNEUROSCI.3232-04.2005 PMC6725315

[CR39] McGuire BA, Stevens JK, Sterling P (1984) Microcircuitry of bipolar cells in cat retina. J Neurosci 4:2920–2938 6502212 10.1523/JNEUROSCI.04-12-02920.1984 PMC6564860

[CR40] Mills SL (1999) Unusual coupling patterns of a cone bipolar cell in the rabbit retina. Vis Neurosci 16:1029–1035 10614585 10.1017/s0952523899166057

[CR41] Münch TA, da Silveira RA, Siegert S, Viney TJ, Awatramani GB, Roska B (2009) Approach sensitivity in the retina processed by a multifunctional neural circuit. Nat Neurosci 12:1308–1316 19734895 10.1038/nn.2389

[CR42] O'Brien J (2019) Design principles of electrical synaptic plasticity. Neurosci Lett 695:4–11 28893590 10.1016/j.neulet.2017.09.003 PMC5843509

[CR43] O'Brien J, Bloomfield SA (2018) Plasticity of retinal gap junctions: roles in synaptic physiology and disease. Annu Rev Vis Sci 4:79–100 29889655 10.1146/annurev-vision-091517-034133

[CR44] O’Brien JJ, Chen X, MacLeish PR, O’Brien J, Massey SC (2012) Photoreceptor coupling mediated by connexin36 in the primate retina. J Neurosci 32:4675–4687 22457514 10.1523/JNEUROSCI.4749-11.2012 PMC3335500

[CR45] Pan F, Mills SL, Massey SC (2007) Screening of gap junction antagonists on dye coupling in the rabbit retina. Vis Neurosci 24:609–618 17711600 10.1017/S0952523807070472 PMC2213422

[CR46] Pereda AE (2016) The variable strength of electrical synapses. Neuron 90:912–914 27253444 10.1016/j.neuron.2016.05.031

[CR47] Peretz A, Degani N, Nachman R, Uziyel Y, Gibor G, Shabat D, Attali B (2005) Meclofenamic acid and diclofenac, novel templates of KCNQ2/Q3 potassium channel openers, depress cortical neuron activity and exhibit anticonvulsant properties. Mol Pharmacol 67:1053–1066 15598972 10.1124/mol.104.007112

[CR48] Pologruto TA, Sabatini BL, Svoboda K (2003) ScanImage: flexible software for operating laser scanning microscopes. Biomed Eng Online 2:13 12801419 10.1186/1475-925X-2-13 PMC161784

[CR49] Raviola E, Gilula NB (1975) Intramembrane organization of specialized contacts in the outer plexiform layer of the retina. A freeze-fracture study in monkeys and rabbits. J Cell Biol 65:192–222 1127010 10.1083/jcb.65.1.192 PMC2111162

[CR50] Strettoi E, Dacheux RF, Raviola E (1990) Synaptic connections of rod bipolar cells in the inner plexiform layer of the rabbit retina. J Comp Neurol 295:449–466 2351763 10.1002/cne.902950309

[CR51] Strettoi E, Raviola E, Dacheux RF (1992) Synaptic connections of the narrow-field, bistratified rod amacrine cell (AII) in the rabbit retina. J Comp Neurol 325:152–168 1460111 10.1002/cne.903250203

[CR52] Strettoi E, Dacheux RF, Raviola E (1994) Cone bipolar cells as interneurons in the rod pathway of the rabbit retina. J Comp Neurol 347:139–149 7798378 10.1002/cne.903470111

[CR53] Szoboszlay M, Lörincz A, Lanore F, Vervaeke K, Silver RA, Nusser Z (2016) Functional properties of dendritic gap junctions in cerebellar Golgi cells. Neuron 90:1043–1056 27133465 10.1016/j.neuron.2016.03.029 PMC4893164

[CR54] Trenholm S, Awatramani GB (2017) Dynamic properties of electrically coupled retinal networks. In: Jing J (ed) Network functions and plasticity. Academic, New York, pp 183–208

[CR55] Trexler EB, Li W, Massey SC (2005) Simultaneous contribution of two rod pathways to AII amacrine and cone bipolar cell light responses. J Neurophysiol 93:1476–1485 15525810 10.1152/jn.00597.2004

[CR56] Tsukamoto Y, Omi N (2015) OFF bipolar cells in macaque retina: type specific connectivity in the outer and inner synaptic layers. Front Neuroanat 9:122 26500507 10.3389/fnana.2015.00122 PMC4594025

[CR57] Tsukamoto Y, Omi N (2017) Classification of mouse retinal bipolar cells: type-specific connectivity with special reference to rod-driven AII amacrine pathways. Front Neuroanat 11:92 29114208 10.3389/fnana.2017.00092 PMC5660706

[CR58] Umino O, Maehara M, Hidaka S, Kita S, Hashimoto Y (1994) The network properties of bipolar-bipolar cell coupling in the retina of teleost fishes. Vis Neurosci 11:533–548 8038127 10.1017/s0952523800002443

[CR59] Vaney DI (1991) Many diverse types of retinal neurons show tracer coupling when injected with biocytin or Neurobiotin. Neurosci Lett 125:187–190 1715532 10.1016/0304-3940(91)90024-n

[CR60] Veruki ML, Hartveit E (2002a) AII (rod) amacrine cells form a network of electrically coupled interneurons in the mammalian retina. Neuron 33:935–946 11906699 10.1016/s0896-6273(02)00609-8

[CR61] Veruki ML, Hartveit E (2002b) Electrical synapses mediate signal transmission in the rod pathway of the mammalian retina. J Neurosci 22:10558–10566 12486148 10.1523/JNEUROSCI.22-24-10558.2002 PMC6758447

[CR62] Veruki ML, Hartveit E (2009) Meclofenamic acid blocks electrical synapses of retinal AII amacrine and ON-cone bipolar cells. J Neurophysiol 101:2339–2347 19279153 10.1152/jn.00112.2009

[CR63] Veruki ML, Mørkve SH, Hartveit E (2006) Activation of a presynaptic glutamate transporter regulates synaptic transmission through electrical signaling. Nat Neurosci 9:1388–1396 17041592 10.1038/nn1793

[CR64] Veruki ML, Oltedal L, Hartveit E (2010) Electrical coupling and passive membrane properties of AII amacrine cells. J Neurophysiol 103:1456–1466 20089813 10.1152/jn.01105.2009

[CR65] Völgyi B, Kovács-Oller T, Atlasz T, Wilhelm M, Gábriel R (2013) Gap junctional coupling in the vertebrate retina: variations on one theme? Prog Retin Eye Res 34:1–18 23313713 10.1016/j.preteyeres.2012.12.002

[CR66] Wu S-N, Jan C-R, Chiang H-T (2001) Fenamates stimulate BK_Ca_ channel activity in the human osteoblast-like MG-63 cells. J Investig Med 49:522–533 11730088 10.2310/6650.2001.33629

[CR67] Yasuda R, Nimchinsky EA, Scheuss V, Pologruto TA, Oertner TG, Sabatini BL, Svoboda K (2004) Imaging calcium concentration dynamics in small neuronal compartments. Sci STKE 219:l5 10.1126/stke.2192004pl5 14872098

[CR68] Zandt B-J, Liu JH, Veruki ML, Hartveit E (2017) AII amacrine cells: quantitative reconstruction and morphometric analysis of electrophysiologically identified cells in live rat retinal slices imaged with multi-photon excitation microscopy. Brain Struct Funct 222:151–182 26951289 10.1007/s00429-016-1206-0 PMC5225199

[CR69] Zandt B-J, Veruki ML, Hartveit E (2018) Electrotonic signal processing in AII amacrine cells: compartmental models and passive membrane properties for a gap junction-coupled retinal neuron. Brain Struct Funct 223:3383–3410 29948192 10.1007/s00429-018-1696-z

[CR70] Zhang A-J, Wu SM (2009) Receptive fields of retinal bipolar cells are mediated by heterogeneous synaptic circuitry. J Neurosci 29:789–797 19158304 10.1523/JNEUROSCI.4984-08.2009 PMC2745915

